# Estimates of past and future time trends in age-specific breast cancer incidence among women in Karachi, Pakistan: 2004–2025

**DOI:** 10.1186/s12889-019-7330-z

**Published:** 2019-07-25

**Authors:** Sidra Zaheer, Nadia Shah, Syed Amir Maqbool, Noor Muhammad Soomro

**Affiliations:** 1 Department of Statistics, University of Karachi, Karachi, Pakistan; 2 School of Public Health, Dow University of Health Sciences, OJHA Campus, Suparco road, Karachi, Pakistan; 3 Department of Clinical Oncology, Karachi Institute of Radiotherapy and Nuclear Medicine, Karachi, Pakistan; 4 Oncology Department, Civil Hospital, Karachi, Pakistan

**Keywords:** Breast cancer incidence, Forecasting, Functional time series model, Log-linear regression model

## Abstract

**Background:**

The current demographic trends indicate that breast cancer will pose an even greater public health concern in future for Pakistan. Details on the incidence, disease severity and mortality in respect of breast cancer are limited and without such data, therefore, future health policies or systems in respect of this disease cannot be strategically planned or implemented. The aim of this study was to examine past trends of age-specific breast cancer incidence rates (2004–2015), and to estimate the future volume of breast cancer cases in Karachi through the year 2025.

**Methods:**

Two statistical methods, namely the functional time series models and the log-linear regression model were used; additionally, their real forecasting efficacy in epidemic time series was also evaluated.

**Results:**

In the past, women aged 60–64 years had the highest overall breast cancer incidence rates, while from 2016 to 2025, large increases in breast cancer rates among women aged 50 to 64 years are expected. The total projected breast cancer incidence will increase by approximately 23.1% in 2020 to 60.7% in 2025. Cases of breast cancer diagnosed in younger women, aged 30–34 years, will increase from 70.7 to 130.6% in 2020 and 2025 relative to 2015.

**Conclusions:**

The breast cancer incidence appeared to have been rising more rapidly among post-menopausal women (aged 55 to 59), while a stable increase in incidence in the youngest age group (15–29 years) of women is expected. The results also infer an expected increase in incidence cases of breast cancer among middle aged women in Karachi, Pakistan. An increase in the number of incident cases of cancer has implications for understanding the health-care needs of growing population and the subsequent demands on health-care system.

**Electronic supplementary material:**

The online version of this article (10.1186/s12889-019-7330-z) contains supplementary material, which is available to authorized users.

## Background

Globally, 2.4 million newly diagnosed female breast cancer cases are predicted on the basis of demographic changes only, accounting for almost 1 in 4 cancer cases among women in 2018 [1, 2]. Although, the incidence of breast cancer in Asia is still lower than that in Western countries, in the modern epoch, the proportional contribution to the global burden of breast cancer is growing rapidly in Asia [2]. This has mainly contributed to recent initiatives such as the establishment of breast cancer registries. The risk of having breast cancer has increased in Pakistan, whereby one in every 9 women in Pakistan has a lifetime risk of being diagnosed with breast cancer [3]. The age-standardized incidence rate of breast cancer in Pakistan is one of the highest among Asian countries [4]. Unfortunately, the rate of deaths due to breast cancer is also higher in the country, due to late diagnosis and delayed referral to appropriate facilities [5]. Hence, early diagnosis and therefore early management of breast cancer can progress the survival rate. The situation is worst in Pakistani women where the number is more than that and no systemic and scientific approach has been employed to combat the situation. The accurate occurrence, number of new cancer cases, death rates, and casualty rate annually for Pakistan are not documented. No comprehensive registries/database existing regarding any disease as well as breast cancer in Pakistan and the only data on hand is hospital based [6].

With the recent advent of modern screening techniques and extended age range for screening, a large disparity in age-adjusted incidence rates has emerged. It has been observed that in Western countries, the rate increases rapidly before menopause, and then increases gradually afterward [1]. The recent increase in breast cancer incidence in Western countries is most apparent in women aged 50 years and older [7]. On the other hand, accumulating data from the developing world suggest substantially higher proportion (47.3%) of all incident breast cancers among pre-menopausal aged compared to older women [8]. Majority of Asian populations display a peak in breast cancer incidence between 40 and 59 years of age; particularly, the peak age-specific incidence rate of breast cancer in India, Korea and Japan are 50–59, 40–49 and 45–54 years respectively [8–10]. Whereas the peak age in Sri Lanka is seen among women aged 60–64 years [11].

The current demographic trends indicate that breast cancer will pose an even greater public health concern in future for Pakistan. Additionally, there is a paucity of information regarding breast cancer in Pakistan [12]. Predictions have been used to advocate the allocation of adequate resources for diagnosis and treatment of disease [13–15]; however, there is little progress on the subject matter in recent times. These call for enhanced focus on research in the area of breast cancer, in order to generating accurate data.

The aim of the present study is to examine past trends of age-specific breast cancer incidence rates among Pakistani women aged > 15 years; and to estimate the future volume of breast cancer cases in Pakistan through the year 2025. Since we are primarily interested in having predictions that are accurate, thus, to achieve this objective, two statistical methods, namely the functional time series (FTS) models and the log-linear regression (LLR) model are applied. Further, their real forecasting efficacy in epidemic time series was also evaluated.

## Methods

### Study settings

Karachi is the largest metropolitan area in Pakistan with a population of approximately 16 million [16]. Breast cancer cases from neighboring districts/ provinces are referred to the major tertiary care facilities established in the city. Jinnah Post Graduate Medical Centre (JPMC), Karachi Institute of Radiotherapy and Nuclear Medicine (KIRAN) hospital and Civil Hospital, Karachi (CHK) are well established in the diagnosis and treatment of cancer patients. Majority of the population of Karachi and interior Sindh, needing cancer treatment, visit one of these centers for their respective cancer treatment. Thus, at present these institutes provide a comprehensive collection of all breast cancer cases in the Sindh region. The data used in this study were collected from these three centers. Although this is a hospital based data, and therefore have some constraints and biases, it is the major source, which can provide essential clinical, administrative and educational information on breast cancer incidence and allow for the estimation of national pattern in the absence of any population based cancer registry in the country [17].

### Study population

A total of 9771 registered diagnosed cases of female breast cancer during the period 2004 to 2015 were included; however, the cases with missing age and duplicate information were excluded in the study. The diagnosis of each patient was according to the international classification of diseases-oncology.

### Estimated age-specific women population, Karachi-Pakistan: 2004–2025

Based on the 1998 census, data on the age-specific female mid-year population of Karachi, Pakistan was obtained from the Pakistan Bureau of Statistics-Sindh, for all six districts of Karachi [16]. Using 1998 census women data, future estimates of age-specific women population for the years 2004–2025 were estimated using Age-Specific Growth Rate (ASGR) [18]. To estimate the future population, following mathematical formula was used:

$$ {P}_t=B\ast {e}^{r\ast t} $$

Here, P = population at a future time, B = population at base census, e = base of natural logarithm (2.71828), r = growth rate, and t = time period in years between future and base population [19].

### Past and future trends of age-specific incidence rates of breast cancer: 2004–2025

Past trends of age-specific women breast cancer incidence rates from 2004 to 2015 were calculated as the number of new cases of breast cancer divided by the corresponding population at risk during a year, expressed as the rates per 100,000 persons per year [4, 20].

For future trends (2016–2025), the age-specific breast cancer incidence rates were estimated using FTS models [4, 21] and LLR model [22, 23]. Past and future estimates were reported for each age group (15–19, 20–24, 25–29, 30–34, 35–39, 40–44, 45–49, 50–54, 55–59, 60–64, 65–69, 70–74, 75+). For each of the forecasting method, we have shown the obtained forecast diagrams which graphically depict the closeness between the original and forecasted incidence rates (Additional file 1: Figure S1 and S2).

### Functional time series (FTS) models

FTS models were recently developed by Hyndman and Ullah 2007 for demographic projections [24]. These models were also applied to the breast cancer mortality rates by Erbas et al. and Yasmeen et al. [21, 25, 26]. To predict future estimates, these models treated incidence rates as a continuous function of age curves. For each curve, the log incidence rates were also obtained separately as follow:

$$ {y}_t\left({v}_i\right)={s}_t\left({v}_i\right)+{\sigma}_t\left({v}_i\right){\varepsilon}_{t,i} $$

Where *y*_*t*_(*v*) = log[*r*_*t*_(*v*)]. Thereafter, the nonparametric smoothing methods were used to obtain smoothed curves from the original log incidence data to reduce the observational error. The smooth curves are treated as functional observations with error.

$$ {s}_t(v)=\mu (v)+\sum \limits_{k=1}^K{\beta}_{t,k}{\varphi}_k(v)+{e}_t(v) $$

Where *μ*(*v*) is the mean log incidence rate across years, *φ*_*k*_(*v*) is a set of orthogonal basis functions, is a univariate time series, and the model error *e*_*t*_(*v*) assumed to be serially uncorrelated i.e. *e*_*t*_(*v*) ~ N(0, *v*(*v*)). Details are mentioned elsewhere [4].

### Log-linear regression (LLR) model

Hakulinen and Dyba (1994) proposed a method for constructing prediction intervals with different models to calculate the number of cases and the age-specific incidence rates by assuming Poisson distribution for counts (number of cases) defined by age and sex [22]. This method can be applied to predict incidence cases based on a model with any functional form of linear predictor or link function [23, 27].

$$ \log E\left[{y}_{it}\right]={\alpha}_i+{\beta}_it $$

Here E[y_it_] is the expected age-specific incidence rate of breast cancer in age group *i*, and time period *t*. Where α_i_ is the baseline age-specific incidence rate at time = 0, and β_i_ is the observed expected annual change in the breast cancer incidence for the age-group *i*.

### Forecast accuracy measures

Further, forecast accuracy as well as performance of both fitted models was evaluated based on the errors obtained using out-of-sample forecast approach, previously applied by Hyndman RJ, and Athanasopoulos [28]. This approach is widely used to evaluate forecasting performance and, in turn, guide the selection of an appropriate forecasting model. The accuracy of forecasts can only be determined by considering how well a model performs on new data that were not used when estimating the model [29]. For this purpose, we divided the observed data into two series. First series (training data) having the observed incidence rates from 2004 to 2011 which is used for estimating the parameters, and forecasting the next set of observations, from 2012 to 2015. Second series (test data) having the observed incidence rates from 2012 to 2015, which is used to test the forecasted observations, produced using the training data.

Let *y*_*t*_ denote the observed actual age-specific incidence rates at time *t (t = 1,2,..,n)*and *f*_*t*_ denote the h-step ahead forecasted age-specific incidence rates of *y*_*t*_. Then the forecast error can be calculated by taking difference between the actual rates in the test data and the h-step ahead forecasts produced using the training data. It is defined as *e*_*t*_ = *y*_*t*_ − *f*_*t*_. A model with small forecast errors (residuals) will give good forecasts. We have used the five forecast accuracy measures defined as:

$$ Mean\kern0.5em Error\ (ME)= mean\left(\ {e}_t\right),\kern0.5em where\ {e}_t={y}_t-{f}_t $$

$$ Mean\ Absolute\ Error\ (MAE)= mean\left(\left|{e}_t\right|\right)=\frac{\sum \limits_{i=1}^n\left|{y}_t-{f}_t\right|}{n} $$

$$ Root\ Mean\ Square\ Error\ (RMSE)=\sqrt{mean\left({e}_t^2\right)} $$

$$ Mean\ Absolute\ Percentage\ Error\ (MAPE)= mean\left(\left|{p}_t\right|\right) $$

Where

$$ {p}_t=100\times {e}_t/{y}_t\operatorname{} $$

$$ Mean\ Absolute\ Scaled\ Error\ (MASE)= MAE/Q $$

Where

$$ Q=\frac{1}{n-1}\sum \limits_{j=2}^n\left|{y}_j-{f}_{j-1}\right| $$

Furthermore, annual changes in the age-specific incidence were calculated using estimated breast cancer cases from FTS model. The changes in incidence were computed as the difference between the predicted incidence for future years (2020 and 2025) and the observed cases for the year 2015.

### Ethical considerations

Formal written approval from hospital administration was obtained regarding use of anonymized and de-identified medical records. Consent to participate was not applicable to this study which was a secondary analysis of de-identified retrospective medical records. Further, none of the researchers had access to identifying information. The study was approved by the Institutional Review Board of the Jinnah Postgraduate Medical Centre, Karachi, Pakistan. (Reference Number: F.2–81/2014-GENL/3434/JPMC).

## Results

Table 1 shows the estimated population (persons per year) of female for 2004 to 2025 by each age group. The proportion of the female population in Karachi was estimated to be increasing in each age group with a slower increase in older ages (≥ 60 years) with the time. The estimates revealed a total female population of over 7 million in year 2017 which nearly equals the newly released provisional figures of census 2017 at national level [16].

**Table 1:** The trends in the age-specific population estimates among women-Karachi Pakistan (2004–2025)

Age-groups (in years)	2004–2005	2006–2007	2008–2009	2010–2011	2012–2013	2014–2015	2016–2017	2018–2019	2020–2021	2022–2023	2024–2025
15–19	1350496	1448418	1553440	1666077	1786881	1916445	2055402	2204436	2364276	2535705	2719564
20–24	1170251	1265186	1367821	1478782	1598744	1728438	1868654	2020244	2184131	2361314	2552870
25–29	923232	988196	1057730	1132158	1211823	1297093	1388364	1486056	1590622	1702547	1822347
30–34	768622	817785	870093	925746	984958	1047958	1114989	1186306	1262184	1342917	1428813
35–39	577176	600731	625247	650764	677321	704964	733734	763678	794844	827283	861044
40–44	527041	550750	575523	601411	628465	656734	686276	717146	749405	783115	818341
45–49	386243	405235	425160	446066	467999	491011	515154	540485	567061	594943	624198
50–54	329060	344551	360771	377754	395536	414156	433651	454066	475441	497821	521256
55–59	223013	237277	252454	268601	285782	304061	323510	344202	366218	389643	414565
60–64	179442	185648	192068	198711	205583	212694	220049	227659	235533	243679	252107
65–69	111856	118536	125615	133116	141065	149488	158415	167874	177899	188523	199780
70–74	75748	78212	80754	83381	86092	88891	91782	94766	97848	101030	104315
75 & above	76361	78216	80116	82062	84055	86097	88189	90330	92525	94772	97074

Estimated population at risk expressed as persons per year

Table 2 shows past and future trends of age-specific breast cancer incidence rates. For past trends (2004–2015), breast cancer rates were considerably lower and stable in ages 15 to 29 years, while the incidence rates for women aged 25 years and older have been increasing overall with slight variations. Women aged 60–64 years had the highest overall breast cancer incidence rates through the years 2004–2015.

**Table 2:** Past and future age-specific breast cancer incidence rate estimates (2004–2025)

Age-groups (in years)	Observed Incidence Rates (2004–2015)						Functional Time Series Model					Log-Linear Regression Model				
	2004–2005	2006–2007	2008–2009	2010–2011	2012–2013	2014–2015	2016–2017	2018–2019	2020–2021	2022–2023	2024–2025	2016–2017	2018–2019	2020–2021	2022–2023	2024–2025
15–19	1.2	0.5	0.4	0.4	0.3	0.3	0.6	0.6	0.6	0.6	0.6	0.2	0.2	0.2	0.0	0.0
20–24	3.1	2.4	2.6	2.6	3.3	4.2	3.6	3.8	4.0	4.1	4.3	4.1	4.4	4.7	5.1	5.6
25–29	10.4	10.8	11.3	10.4	13.9	14.4	14.4	15.2	16.2	17.0	17.8	14.4	15.3	16.2	17.3	18.3
30–34	23.1	21.2	23.7	21.8	30.7	33.1	38.4	40.9	43.5	46.2	48.9	31.9	34.5	37.5	40.6	43.9
35–39	62.8	50.3	55.8	48.6	69.9	81.4	75.7	81.0	86.4	92.0	97.6	74.3	78.9	83.8	89.0	94.4
40–44	72.2	70.5	80.4	74.5	99.1	134.0	119.3	127.8	136.7	145.7	154.9	129.5	146.0	164.6	185.6	209.3
45–49	90.6	90.9	100.8	95.9	121.8	154.2	159.5	171.1	182.8	194.7	206.8	146.7	162.1	179.2	198.0	218.9
50–54	102.7	114.2	117.7	108.0	143.8	169.4	187.7	201.0	214.4	228.0	241.6	154.9	167.9	181.9	197.0	213.5
55–59	94.7	105.5	130.5	103.7	149.0	186.6	198.3	211.8	225.3	238.8	252.2	189.3	213.4	240.7	271.4	306.0
60–64	114.8	122.8	155.3	141.9	156.6	202.1	191.3	203.6	215.7	227.8	239.7	206.1	227.8	251.7	278.2	307.5
65–69	107.1	109.6	111.4	88.8	116.2	156.5	170.9	180.9	190.8	200.6	210.2	139.5	148.2	157.3	167.1	177.4
70–74	95.1	99.5	99.2	129.4	155.7	152.8	142.9	150.4	157.8	164.8	171.9	177.1	198.8	223.3	250.8	281.6
75 & above	23.6	61.3	72.4	109.6	85.7	113.8	124.7	130.9	136.7	142.4	148.0	150.4	185.2	228.0	280.8	345.7

Age-specific breast cancer incidence rates per 100,000 persons per year

For the next 10 years and according to the predictions of the FTS model, predictions from FTS model, large increases in breast cancer rates among women aged 50 to 64 years are expected, while low increase in rates are expected among women of other ages. Specifically, the rate for women aged 55–59 years showed the highest overall breast cancer incidence, followed by women aged 50–54 and 60–64 years. Whereas LLR model predictions revealed that women older than age 75 years are at higher risk of breast cancer, followed by those women aged between 55 and 64 years. Dramatic decrease in rates was predicted among women aged 30–39 years, and rates should be considerably stable among ages 15–29 years. Future incidence trends with comparison to past trends were also depicted in Additional file 1.

Past and future burden of age-specific breast cancer cases for the years 2004 to 2025 showed in Table 3. Future estimates of breast cancer cases were computed by multiplying estimates of breast cancer incidence rates and risk population for each age group. For past trends, during the study period (2004–2015), there were large increases in annual age-specific breast cancer cases for middle ages, considerable increases were observed among women aged 40–54 years.

**Table 3:** Past and future predicted age-specific breast cancer incidence estimates (2004–2025)

Age-groups (in years)	Observed Incidence (2004–2015)						Functional Time Series Model					Log-Linear Regression Model				
	2004–2005	2006–2007	2008–2009	2010–2011	2012–2013	2014–2015	2016–2017	2018–2019	2020–2021	2022–2023	2024–2025	2016–2017	2018–2019	2020–2021	2022–2023	2024–2025
15–19	8	4	3	4	3	3	6	6	6	7	8	2	2	2	2	0
20–24	18	15	18	19	27	36	35	39	43	49	55	38	44	52	61	71
25–29	48	53	59	59	84	93	101	113	128	144	163	100	114	130	147	167
30–34	89	87	103	101	152	173	214	243	275	310	350	178	205	236	272	315
35–39	181	151	174	158	237	287	278	310	343	380	420	273	301	333	368	406
40–44	190	194	231	224	312	440	410	459	512	571	634	444	524	617	727	857
45–49	175	184	214	214	286	379	411	462	518	580	645	378	438	509	590	683
50–54	169	197	212	204	285	351	407	457	510	567	630	336	382	432	491	556
55–59	106	125	164	139	213	284	321	365	413	466	523	306	367	441	529	635
60–64	103	114	149	141	161	215	211	232	254	278	302	227	259	296	339	387
65–69	60	65	70	59	82	117	136	152	170	189	210	111	124	140	157	177
70–74	36	39	40	54	67	68	65	71	77	83	90	81	94	110	127	147
75 & above	9	24	29	45	36	49	55	59	63	67	71	66	83	106	133	168
Age-specific breast cancer incidence cases per 100,000 persons per year.																

Similar trends were observed for predicted cases for the next 10 years (2016–2025). The combined observed cases had increased from 1192 cases in 2004–2005 to 2495 cases in 2014–2015 (more than twice), and the expected cases of breast cancer are predicted to increase 4101 (FTS) / 4569 (LLR) cases in 2024–2025. For the next 10 years (from 2014 to 2015), future average annual breast cancer incidence increased 1.6 fold-FTS (1.8 fold-LLR). Absolute numbers of breast cancer cases for each age group were predicted to increase except among younger ages, 15 to 19 years. Highest breast cancer burden was observed for women aged 40–44 years, predicted breast cancer cases increased from 440 in 2014–15 to 857 in 2024–2025 (1.9 fold increase-LLR). Increasing cases were noticeable for women aged 40 years and above, incidence for women aged 40–59 years have increased to 1454 in 2014–15 to 2432 (2731-LLR) cases by 2024–2025. Data showed in detail in Table 3.

Forecast accuracy as well as the comparison between the two models fitted to predict the incidence rates, are shown in Table 4, indicating that FTS model is giving small absolute errors as compared to measures computed with the LLR model. This shows that FTS model performed better and gives good forecasts than the other fitted model. Further, forecasting performance across ages for both models was also assessed, and results showed similar forecast variations for ages 20 to 59, however, absolute differences in both models were observed in predicted values for older ages (60 and above). [See Additional file 1: Table S1 and S2].

**Table 4:** Accuracy measures of the models computed from test data

Model	ME	MAE	RMSE	MAPE	MASE
FTS	12.3	13.5	17.3	29.2	1.0
LLR	6.7	15.7	21.5	33.7	1.2

*FTS* Functional time series, *LLR* Log-linear regression, *ME* Mean error, *MAE* Mean absolute error, *RMSE* Root mean square error, *MAPE* Mean absolute percentage error, *MASE* Mean absolute scaled error

Table 5 presents the annual changes in breast cancer incidence in 2020 and 2025 relative to 2015, among all age groups. Between 2015 and 2020, the largest increase in breast cancer incidence is expected among younger women aged less than 34 years (70.7% for women aged 30–34 years). Whereas, women aged 30–34 years may be responsible for a 130.6% increase in incidence by 2025. The incidence for all ages is predicted to increase from 1309 in 2015 to 1611 (23.1% increased) in 2020 and 2103 in 2025, which is 60.7% higher than the incidence in 2015.

**Table 5:** FTS-% change in age-specific breast incidence 2015–2025

Age-groups	Annual changes in breast cancer incidence compared with 2015				
	2015	2020		2025	
	Cases	Difference	%	Difference	%
15–19	2	1	57.8	2	99.0
20–24	16	5	31.5	12	76.7
25–29	41	21	51.7	43	104.5
30–34	78	55	70.7	102	130.6
35–39	150	17	11.6	65	43.6
40–44	231	18	7.9	94	40.7
45–49	209	43	20.5	122	58.5
50–54	190	58	30.5	133	69.9
55–59	153	47	30.7	116	75.8
60–64	116	8	7.0	38	33.0
65–69	60	23	37.6	48	79.5
70–74	38	0	−0.4	8	20.1
75 & above	25	6	24.4	11	45.8
Total	1309	302	23.1	794	60.7

Age-specific breast cancer cases per 100,000 persons per year

## Discussion

This paper presents past and future estimates of breast cancer incidence rates and expands our understanding of age and time related differences in incidence rate of breast cancer among women of Karachi, Pakistan. The cancer statistics from Karachi potentially reflect national level picture, because it is the only city of Pakistan which is composed of considerable population from various ethnic groups. Therefore, in the absence of a national level cancer registration process, medical records from hospitals in Karachi can be considered as highly representative of Pakistani population [30].

Prior studies from the region have highlighted increase in breast cancer burden among women in Pakistan [17, 30–32], however none of these studies applied statistical modeling and moreover, these studies were based on cross-sectional data. In this study, we assessed two models and report for the first time comparison of these approaches to incidence prediction of breast cancer in Pakistani females for the period of 2016–2025, as well as with the focus on testing the reliability of the methods. We show increasing breast cancer trend during 2004 to 2015, and we expect the trend will continue to rise in the future, particularly during the period 2016–2025. However, the age-specific breast cancer incidence rates are found heterogeneous. Between 2004 and 2015, overall incident rates were highest among women aged 60–64 years while from 2016 to 2025 large increases in breast cancer rates among women aged 55 to 64 years and women older than age 75 years are expected. Nevertheless, the overall breast cancer incidence appeared to be rising more rapidly among post-menopausal women, while a stable increase in incidence in youngest age group of women aged 15–29 years was observed. The results also infer an increase in incidence rate of elderly breast cancer similar to the patterns observed among women in developed countries [1, 8, 33].

In the present study, we expect the number of breast cancer cases to increase by 60.7% (approximately to 2103 to be diagnosed by 2025). Although, past trend of breast cancer cases among elderly women (aged 65 and above) were rare compared to women aged 60 to 64 years, but these would become common in 2021–2025. The volume of pre-menopausal cases (aged 40 to 44 years) would increase from 7.9% in 2020 to 40.7% in 2025. Compared to pre-menopausal women, the projected increase in breast cancer cases among post-menopausal women (aged 55 to 59) is relatively higher (from 30.7 to 75.8% in 2020 and 2025). Even though, the incidence among younger age groups is very low, but these results may reflect less screening efforts targeting this age group. Therefore, the reported breast cancer burden may be underestimated. The lack of specialized care units and the weak data bases in Pakistan contribute to the reported lower incidences [34]. Many women would have to be screened to find the cases, thus we suggest greater awareness on early signs of breast cancer among younger women and education on breast self-examination to realize early detection among the younger age groups.

The predicted values from the applied models showed gradual and uniform increase in the incidence of breast cancer over the study period (i.e. during 2004–2025) in Pakistan (See Additional file 1: Figure S1 and S2).

Breast cancer incidence remain highest in the age group of 60 to 64 during 2004 to 2015 but the FTS estimates showed the shift in maximum rate of incidence in the age group of 55 to 59. While, according to LLR predictions same age group (i.e. 55 to 59 years) will experience highest incidence till 2025. Moreover, FTS model showed small absolute errors than other predicted model. This indicates that the forecasting performance of FTS model in predicting incidence rates was better compared to the LLR model. Earlier epidemiological studies from Asian and Western countries have presented notable difference in peak incidence, which was between 40 to 50 years in Asian women whereas the peak incidence was between 60 to 70 years in Western women [35–37]. We found not only increase in age-specific incidence rate in Pakistani women but also predicted the peak age of onset continuing in post-menopausal women which is somewhat similar to the incidence reported from other Western type data [9, 35]. The reduction in variation observed in peak onset age between western countries and our population is most likely attributable to rise in urbanized and westernized culture in our society [15, 36]. However, we need more researches to determine similarities and differences in breast cancer among women from Asian countries to compare with diverse global populations with different geographic, racial/ethnic, genetic, lifestyle and socioeconomic backgrounds.

The demographic component underlying the increasing future burden of breast cancer is likely to continue as Pakistan population is expected to increase, with the largest increases expected in population aged 15–64. Although, the population under 40 years, except the young adolescent age, is rising at a slower rate; high fertility will remain one of the key forces contributing to further population growth. In contrast, the population of 65–74 years is small but growing. As a result, rapid population aging and increase in working age women is now taking place [38]. Under such conditions, breast cancer incidence rate and the age structure of women with breast cancer will change with time.

With this, the rise in incidence of breast cancer also reflects noticeable changes in distribution of risk factors associated with shifts in life style and changing socioeconomic development [35, 36]. There is evidence that increase in adoption of cancer associated lifestyle choices including smoking, physical inactivity, and “westernized” diets are emerging risk factors for breast cancer incidence in the region [39]. Another study reported illiteracy, cultural and economic hindrance as reasons for late consultation of medical physician [40]. These factors have important implications for stage presentation at diagnosis and highlight an immediate attention for early detection strategies and cheap access to healthcare provision. In addition, accurate projections of age-specific breast cancer incidence may prove to be helpful, particularly for the planning and implementation of mammographic screening programs. In this regard, both models performed rather similarly, but the FTS model worked slightly better than LLR model. This could be probably because the FTS models take age as a continuous function, which allows capturing the subtle patterns of variation between years. Thus; reduced the observational error by use of smoothed data before beginning the estimation of basic functions [21, 26]. However, the second approach, LLR model, is used with the assumption that the observations either age or period specific incidence cases must be independent and follow Poisson distributions [22]. Since the actual projection period in the current study is short (10 years, 2016–2025), we used only the log-linear regression model, rather than models more appropriate for increasing rates that specify an identity link function [27].

This study has a number of important strengths. First, we have presented an alternative modelling approach for estimation of breast cancer incidence, thereby enhancing the accuracy of the prediction intervals for future incidence rates. Further, these models will be most useful for modeling and projecting the future trends of other cancers as well, for which there has been very little advancement in treatment and opportunities for prevention, early detection, or both, are few. While we have made an attempt to provide a robust estimation of past and future age-specific breast cancer incidence, we are aware that there are some limitations to this study. First, our study lacks inclusion of birth cohort effect, breast cancer subtype information and other important risk factors such as screening and treatment options associated with hormone replacement therapy that may influence age-related changes in incidence. Currently FTS model do not incorporate such effects, but smoothing process used in FTS modelling may reduce the variation attributable to such effects [26]. It is likely that improving prediction models will require inclusion of additional known risk factors which may play a large role in the surveillance, treatment, and survival outcomes of this disease [21]. Future studies are suggested to incorporate these detail assessments to provide a more comprehensive understanding of breast cancer temporal patterns in Pakistan. Secondly, there is a possibility/limitation that there are more breast cancer cases in the city than described herein. Those could potentially include patients who do not have access to hospitals and/or that are diagnosed at other health facility. It could happen that these patients were the most economically disadvantaged. However, our data is based on the largest possible registries of the city, thus serving as the major source in breast cancer data in Karachi, and thereby allowing better estimates of national pattern in the absence of any population based cancer registry in the country [17, 41]. Therefore we recommend that the health system should emphasize on gathering nationwide formal cancer registry data to facilitate efficient planning of research to reduce breast cancer burden as well planning of future cancer services.

## Conclusions

In conclusion, this analysis demonstrates increase in the number of incident cases of breast cancer in Pakistani women. The breast cancer incidence appeared to be rising more rapidly among post-menopausal women, while a stable increase in incidence among youngest age group of women are expected. The age-specific patterns of breast cancer incidence in Pakistan are in line with other countries in Asia, however the shift in maximum rate of breast cancer incidence in the age group of 55 to 59 was observed.

## Additional file

Additional file 1 : Microsoft Word file (doc) providing details of the supplementary figures and tables. (DOCX 2520 kb)

## Data Availability

Data included in the current study are not publicly available to ensure confidentiality of the patients but are available from the corresponding author on reasonable request. However, all data generated or analysed during this study are included in this published article.

## Abbreviations

ASGR: Age-Specific Growth Rate
CHK: Civil Hospital Karachi
FTS: Functional Time Series
JPMC: Jinnah Post Graduate Medical Centre
KIRAN: Karachi Institute of Radiotherapy and Nuclear Medicine
LLR: Log-Linear Regression
MAE: Mean Absolute Error
MAPE: Mean Absolute Percentage Error
MASE: Mean Absolute Scaled Error
ME: Mean Error
RMSE: Root Mean Square Error
