# Supplementary material for: Estimates of past and future time trends in age-specific breast cancer incidence among women in Karachi, Pakistan: 2004–2025
Source: BMC Public Health. 2019 Jul 25;19:1001. doi: 10.1186/s12889-019-7330-z (PMC6659231; doi:10.1186/s12889-019-7330-z)
Supplement: Supplementary file 1 — : Microsoft Word file (doc) providing details of the supplementary figures and tables. (DOCX 2520 kb) [file 12889_2019_7330_MOESM1_ESM.docx]

**Additional file 1**


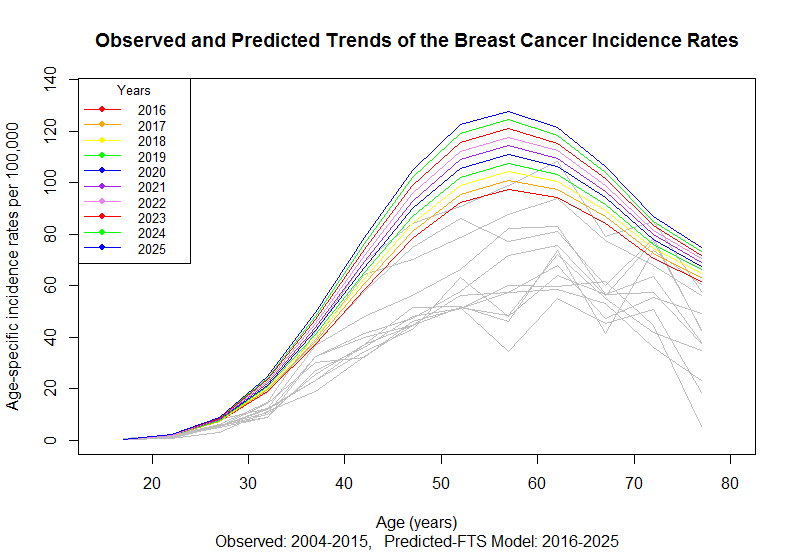


**Fig. S1: Past and future breast cancer incidence trends using FTS model.** Ten-year predictions for breast cancer incidence rates using FTS model: Gray curves show the observed incidence from 2004-2015.


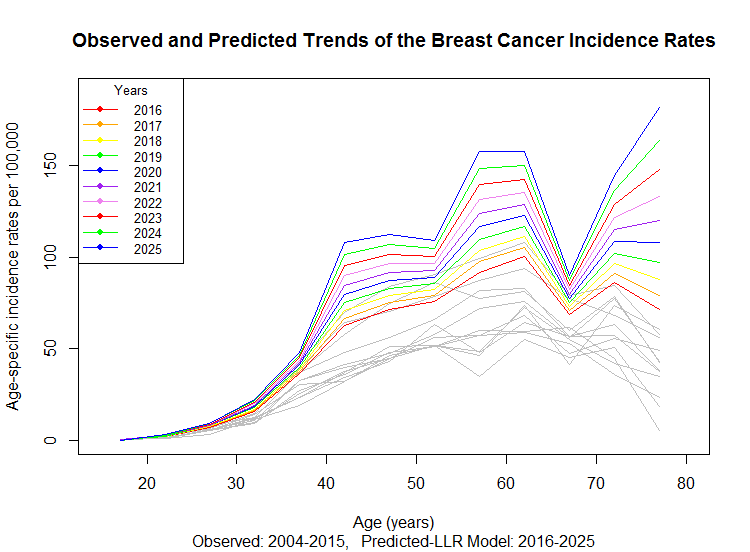


**Fig. S2: Past and future breast cancer incidence trends using LLR model.** Ten-year predictions for breast cancer incidence rates using LLR model: Gray curves show the observed incidence from 2004-2015.

| **Table S1: Forecast accuracy across ages (15 to 75+) for years 2012 to 2015-FTS model** | | | | | | |  |
| --- | --- | --- | --- | --- | --- | --- | --- |
|  |  |  |  |  |  |  |  |
| **Age-groups** | **ME** | **MAE** | **RMSE** | **MAPE** | | **MASE** |  |
| 15-19 | 0.0 | 0.1 | 0.1 | 44.2 | | - |  |
| 20-24 | 0.7 | 0.7 | 0.8 | 35.4 | | 0.5 |  |
| 25-29 | 2.6 | 2.6 | 2.8 | 35.4 | | 1.3 |  |
| 30-34 | 4.4 | 4.9 | 5.7 | 27.6 | | 1.8 |  |
| 35-39 | 15.3 | 15.3 | 15.7 | 39.8 | | 1.0 |  |
| 40-44 | 23.2 | 23.2 | 25.6 | 37.4 | | 4.8 |  |
| 45-49 | 22.9 | 22.9 | 26.9 | 29.8 | | 2.5 |  |
| 50-54 | 22.9 | 22.9 | 26.3 | 26.9 | | 1.5 |  |
| 55-59 | 22.4 | 22.4 | 24.9 | 25.6 | | 1.4 |  |
| 60-64 | 26.0 | 26.0 | 28.9 | 27.7 | | 2.5 |  |
| 65-69 | 5.9 | 9.6 | 11.4 | 13.1 | | 2.4 |  |
| 70-74 | 19.0 | 19.0 | 19.9 | 24.2 | | 1.3 |  |
| 75+ | -4.9 | 5.8 | 7.6 | 13.3 | | 2.0 |  |
| **Mean** | 12.3 | 13.5 | 15.1 | 29.2 | | 1.9 |  |
| FTS: Functional time series | | | | | | | |
| ME: Mean error | | | | |  |  |  |
| MAE: Mean absolute error | | | | |  |  |  |
| RMSE: Root mean square error | | | | |  |  |  |
| MAPE: Mean absolute percentage error | | | | |  |  |  |
| MASE: Mean absolute scaled error | | | | |  |  |  |

| **Table S2: Forecast accuracy across ages (15 to 75+) for years 2012 to 2015-LLR model** | | | | | | |
| --- | --- | --- | --- | --- | --- | --- |
|  |  |  |  |  |  |  |
| **Age-groups** | **ME** | **MAE** | **RMSE** | **MAPE** | **MASE** | |
| 15-19 | 0.1 | 0.1 | 0.1 | 35.3 | - | |
| 20-24 | 0.8 | 0.8 | 0.9 | 36.5 | 0.1 | |
| 25-29 | 2.0 | 2.0 | 2.2 | 26.4 | 1.0 | |
| 30-34 | 4.3 | 4.6 | 5.5 | 25.7 | 0.6 | |
| 35-39 | 17.0 | 17.0 | 17.6 | 44.3 | 1.0 | |
| 40-44 | 20.8 | 20.8 | 23.3 | 33.2 | 2.8 | |
| 45-49 | 18.5 | 19.8 | 22.7 | 26.2 | 2.0 | |
| 50-54 | 21.2 | 21.2 | 24.5 | 24.9 | 11.4 | |
| 55-59 | 24.3 | 24.3 | 26.1 | 28.1 | 11.0 | |
| 60-64 | 4.7 | 7.9 | 9.9 | 8.1 | 13.8 | |
| 65-69 | 22.2 | 22.2 | 24.9 | 30.8 | 1.3 | |
| 70-74 | 6.0 | 8.4 | 8.7 | 10.8 | 4.3 | |
| 75+ | -54.5 | 54.5 | 57.2 | 107.4 | 0.6 | |
| **Mean** | 6.7 | 15.7 | 17.2 | 33.7 | 4.1 | |
| LLR: Log-linear regression | | | | | | |
| ME: Mean error | | | | | |  |
| MAE: Mean absolute error | | | | | |  |
| RMSE: Root mean square error | | | | | |  |
| MAPE: Mean absolute percentage error | | | | | |  |
| MASE: Mean absolute scaled error | | | | | |  |
